# Both Pharmacokinetic Variability and Granuloma Heterogeneity Impact the Ability of the First-Line Antibiotics to Sterilize Tuberculosis Granulomas

**DOI:** 10.3389/fphar.2020.00333

**Published:** 2020-03-24

**Authors:** Joseph M. Cicchese, Véronique Dartois, Denise E. Kirschner, Jennifer J. Linderman

**Affiliations:** ^1^Department of Chemical Engineering, University of Michigan, Ann Arbor, MI, United States; ^2^Public Health Research Institute, New Jersey Medical School, Rutgers, The State University of New Jersey, Newark, NJ, United States; ^3^Center for Discovery and Innovation, Hackensack Meridian Health, Nutley, NJ, United States; ^4^Department of Microbiology and Immunology, University of Michigan Medical School, Ann Arbor, MI, United States

**Keywords:** agent-based model, pharmacokinetic/pharmacodynamic (PK/PD) model, multi-scale model, isoniazid, rifampin, tissue distribution

## Abstract

Tuberculosis (TB) remains as one of the world's deadliest infectious diseases despite the use of standardized antibiotic therapies. Recommended therapy for drug-susceptible TB is up to 6 months of antibiotics. Factors that contribute to lengthy regimens include antibiotic underexposure in lesions due to poor pharmacokinetics (PK) and complex granuloma compositions, but it is difficult to quantify how individual antibiotics are affected by these factors and to what extent these impact treatments. We use our next-generation multi-scale computational model to simulate granuloma formation and function together with antibiotic pharmacokinetics and pharmacodynamics, allowing us to predict conditions leading to granuloma sterilization. In this work, we focus on how PK variability, determined from human PK data, and granuloma heterogeneity each quantitatively impact granuloma sterilization. We focus on treatment with the standard regimen for TB of four first-line antibiotics: isoniazid, rifampin, ethambutol, and pyrazinamide. We find that low levels of antibiotic concentration due to naturally occurring PK variability and complex granulomas leads to longer granuloma sterilization times. Additionally, the ability of antibiotics to distribute in granulomas and kill different subpopulations of bacteria contributes to their specialization in the more efficacious combination therapy. These results can inform strategies to improve antibiotic therapy for TB.

## Introduction

Tuberculosis (TB) continues to be one of the world's deadliest infectious diseases, leading to the death of 1.3 million people in 2017, about 2–3 people per minute (Global tuberculosis report, 2018). Caused by infection with the pathogen *Mycobacterium tuberculosis* (Mtb), TB most commonly presents as a pulmonary disease in adults when individuals inhale aerosolized Mtb transmitted by other infected individuals. The immune response in the lungs leads to the formation of multiple lesions called granulomas, collections of immune cells that act to contain the infection both immunologically and physically but also present a barrier to antibiotic diffusion and delivery (Ramakrishnan, 2012; Dartois, 2014; Pienaar et al., 2015a). Understanding penetration and distribution of antibiotics in granulomas is critical to understanding how best to treat TB.

The current recommended regimen to treat active, drug-susceptible TB disease requires up to six months of multiple antibiotics (Nahid et al., 2016). For the first two months, patients take daily doses of isoniazid (INH, H), rifampin (RIF, R), ethambutol (EMB, E), and pyrazinamide (PZA, Z), referred to as HRZE. Each of these antibiotics has side effects associated with their use that, together with the lengthy treatment duration, make it difficult for patients to properly adhere to the regimen (Yee et al., 2003; Munro et al., 2007). Efforts such as directly observed therapy (DOT) attempt to increase patient adherence but are not tractable on a global scale (Steffen et al., 2010; McLaren et al., 2016). Emergence of multidrug-resistant (defined as resistant to INH and RIF) and extensively drug-resistant TB (resistant to INH, RIF, and a second-line injectable) further complicates treatment (Global tuberculosis report, 2018). There is a need for improved antibiotic therapy for TB and to understand what causes treatment failure.

There are two key factors outside of drug resistance that have been identified as contributing to drug failure in TB: pharmacokinetic (PK) variability (acting at population scale) and granuloma heterogeneity (acting at the host scale). How these factors interact to affect both the rate and extent of sterilization during treatment is not well-understood. PK variability is defined as differences in plasma antibiotic exposure, typically measured as variability in plasma area under the curve (AUC) measurements. Population PK models can help determine appropriate dosing of TB antibiotics and represent this variability based on distributions of PK model parameters (Zhu et al., 2004; Jonsson et al., 2011; Denti et al., 2015; Lalande et al., 2015). These distributions can be related to natural differences in populations through covariates such as weight, age, or overall health. Additionally, PK variability can be due to differences at the genetic scale, such as in N-acetyltransferase 2 involved in the metabolism of INH (Blum et al., 1991; Kinzig-Schippers et al., 2005). This PK variability can lead to poor exposure in granuloma lesions, reducing the amount of time antibiotic concentrations are above therapeutic thresholds during therapy (Strydom et al., 2019).

Host-scale heterogeneity encompasses host-level variations in granuloma number, size, and composition. Granuloma size and composition can lead to slower diffusion of antibiotics, spatial gradients of concentration, and underexposure at the host tissue scale (Dartois, 2014; Pienaar et al., 2015a; Prideaux et al., 2015; Sarathy et al., 2016). Granuloma composition can affect how antibiotics accumulate or fail to accumulate within lesions (Dartois, 2014). For example, EMB's clinical efficacy may partially be explained by its ability to accumulate in cellular regions of the granuloma (Zimmerman et al., 2017). Structural differences in lesions affect the sterilizing ability of PZA, as shown in different strains of mice (Irwin et al., 2016). Caseous regions of the granuloma may also harbor bacteria that are phenotypically more tolerant, and may be less accessible, to many TB antibiotics (Sarathy et al., 2018).

Capturing both PK variability at the population scale and granuloma heterogeneity at the host scale in a computational model can help predict granuloma sterilization and design antibiotic regimens. Our group previously developed a computational model that incorporates the host formation of granulomas and antibiotic PK to predict the sterilization of granulomas using different regimens with INH and RIF (Pienaar et al., 2015a; Pienaar et al., 2015b). Using this multi-scale, systems pharmacology model, we have also highlighted major differences between members of the fluoroquinolone drug class and simulated TB therapy with development of antibiotic resistance (Pienaar et al., 2017; Pienaar et al., 2018). Using this computational framework provides a way to include both PK variability and granuloma heterogeneity to predict whether a treatment can achieve granuloma sterilization in primary, pulmonary TB in adults.

Here we use our hybrid, multi-scale agent-based model to capture PK variability and granuloma heterogeneity and to simulate antibiotic treatment of primary, lung granulomas. For the first time with this model, we simulate treatment based on human PK and with the combination of the four first-line antibiotics used to treat TB: INH, RIF, EMB, and PZA. We also present a sequential calibration scheme that captures spatial distributions of antibiotics within granulomas and known PK variability that exists across the population scale and at the host scale within granulomas. Using this highly detailed model, we discuss the role of first-line antibiotics (HRZE) in sterilizing granulomas and how PK variability and granuloma heterogeneity impact distributions of sterilization times.

## Methods

### Computational Model of Granuloma Formation and Function

*GranSim* is a well-established hybrid, multi-scale computational model that produces the emergent behavior of granuloma formation in Mtb infection (Segovia-Juarez et al., 2004; Fallahi-Sichani et al., 2011; Cilfone et al., 2013; Linderman et al., 2015). Briefly, this agent-based model simulates immune cell movement and interactions, and bacterial growth on a spatial grid representing an area of lung tissue. The immune cell agents, such as different classes of macrophages and T cells, move in response to chemokine gradients and interact with each other according to immunology-derived rules to activate or deactivate immune cells/responses. Bacteria in the model are simulated as individual agents, and they exist in three distinct subpopulations: extracellular replicating, extracellular non-replicating, or intracellular (inside macrophages). The effective growth rate of bacteria in these three subpopulations is influenced by extracellular or intracellular location and availability of nutrients and oxygen (Pienaar et al., 2016). Non-replicating Mtb represent bacteria trapped within caseum, which presents hypoxic conditions with limited nutrient resources (Nathan and Barry III, 2015; Sarathy et al., 2018). A more detailed explanation of *GranSim* and the simulation rules and assumptions can be found online (http://malthus.micro.med.umich.edu/GranSim/). *GranSim* simulates lung granulomas that form due to primary, pulmonary infection in adults and captures a wide diversity of granulomas through variations in host immune system parameters and stochastic events in the agent-based model. The boundary of the granuloma is defined by regions of high cell density, and outlines of the granuloma are drawn to enclose these regions. Parameters that were varied to generate our library of heterogeneous granulomas are listed in **Table 1**.

**Table 1:** Host immune parameters for *in silico* granulomas. Timestep units represent 10-min time steps in the agent-based simulation. Parameter values based on previously published work (Pienaar et al., 2015a).

**Parameter definition**	**Units**	Low CFU Granulomas		High CFU Granulomas	
		**Min**	**Max**	**Min**	**Max**
# immune cell deaths causing compartment caseation		6	10	6	10
Time to heal caseated compartment	Timesteps	909	1,365	901	1,398
TNF threshold for causing immune cell apoptosis	Molecules	690	1,035	690	1,200
Rate constant for TNF-induced apoptosis	1/s	1.36e-6	2.04e-6	1.00e-6	2.00e-6
Minimum chemokine concentration to induce chemotaxis	Molecules	0.27	0.41	0.27	0.41
Maximum chemokine concentration to induce chemotaxis	Molecules	392	588	392	588
Initial density of macrophages	Fraction of grid compartments	0.019	0.029	0.019	0.029
Time between resting macrophage movements	Timesteps	4	6	4	6
Time between active macrophage movements	Timesteps	15	23	15	23
Time between infected macrophage movements	Timesteps	169	255	169	255
TNF threshold to induce NFkB activation	Molecules	42.8	64.1	35.1	65.0
Rate constant for NFkB activation	1/s	6.77e-6	1.01e-5	6.00e-6	1.00e-5
Probability resting macrophage kills extracellular Mtb		0.0738	0.111	0.0738	0.111
Killing probability adjustment for resting macrophages with NFkB activation		0.129	0.194	0.129	0.194
# bacteria to cause NFkB activation		236	354	236	354
# bacteria for macrophage to become chronically infected		12	18	12	18
# bacteria to cause macrophage to burst		19	29	19	29
# bacteria activated macrophage can phagocytose		3	5	3	5
Probability activated macrophage will will heal a caseated compartment		0.00459	0.00687	0.00459	0.00687
Probability a T-cell will move to same compartment as a macrophage		0.0367	0.0550	0.0251	0.0550
Probability IFNγ producing T-cell induces Fas/FasL apoptosis		0.0293	0.0439	0.0290	0.0440
Probability IFNγ producing T-cell also produces TNF		0.0514	0.0770	0.0510	0.0779
Probability cytotoxic T-cell kills macrophage		0.00505	0.0121	0.00806	0.0121
Probability cytotoxic T-cell kills a macrophage and all its intracellular bacteria		0.619	0.928	0.611	0.920
Probability regulatory T-cell deactivates macrophage		0.00584	0.00876	0.00580	0.00880
Time when T-cell recruitment begins	Timesteps	3,225	4,722	3,225	4,397
Time delay after T-cell recruitment begins until maximal recruitment rate	Timesteps	650	976	650	849
Macrophage maximal recruitment probability		0.0241	0.0361	0.0240	0.0500
Macrophage threshold for recruitment by chemokines	Molecules	0.641	0.960	0.640	0.960
Macrophage threshold for recruitment by TNF	Molecules	0.00859	0.0129	0.00851	0.0130
Macrophage half sat for recruitment by TNF	Molecules	1.22	1.82	1.21	1.83
Macrophage half sat for recruitment by chemokine	Molecules	1.68	2.52	1.68	2.52
IFNγ producing T-cell maximal recruitment probability		0.0484	0.0726	0.0300	0.0620
IFNγ producing T-cell threshold for recruitment by chemokine	Molecules	0.0535	0.0802	0.0530	0.0800
IFNγ producing T-cell threshold for recruitment by TNF	Molecules	1.01	1.51	1.00	1.51
IFNγ producing T-cell half sat for recruitment by TNF	Molecules	1.22	1.82	1.21	1.82
IFNγ producing T-cell half sat for recruitment by chemokine	Molecules	1.64	2.46	1.63	2.45
Probability a IFNγ producing T-cell is cognate		0.0437	0.0655	0.0201	0.0650
Cytotoxic T-cell maximal recruitment probability		0.0370	0.0554	0.0370	0.0550
Cytotoxic T-cell threshold for recruitment by chemokine	Molecules	3.55	5.32	3.54	5.32
Cytotoxic T-cell threshold for recruitment by TNF	Molecules	0.920	1.38	0.922	1.38
Cytotoxic T-cell half sat for recruitment by TNF	Molecules	0.715	1.07	0.711	1.07
Cytotoxic T-cell half sat for recruitment by chemokine	Molecules	5.24	7.86	5.25	7.85
Probability a cytotoxic T-cell is cognate		0.0414	0.0620	0.0410	0.0619
Regulatory T-cell maximal recruitment probability		0.0246	0.0369	0.0242	0.0618
Regulatory T-cell threshold for recruitment by chemokine	Molecules	2.03	3.04	2.02	3.04
Regulatory T-cell threshold for recruitment by TNF	Molecules	1.65	2.47	1.65	2.47
Regulatory T-cell half sat for recruitment by TNF	Molecules	2.00	3.00	2.00	3.00
Regulatory T-cell half sat for recruitment by chemokine	Molecules	1.23	1.84	1.22	1.84
Probability a regulatory T-cell is cognate		0.0400	0.0600	0.0401	0.0600

### *In Silico* Granuloma Library

**Figure 1:**
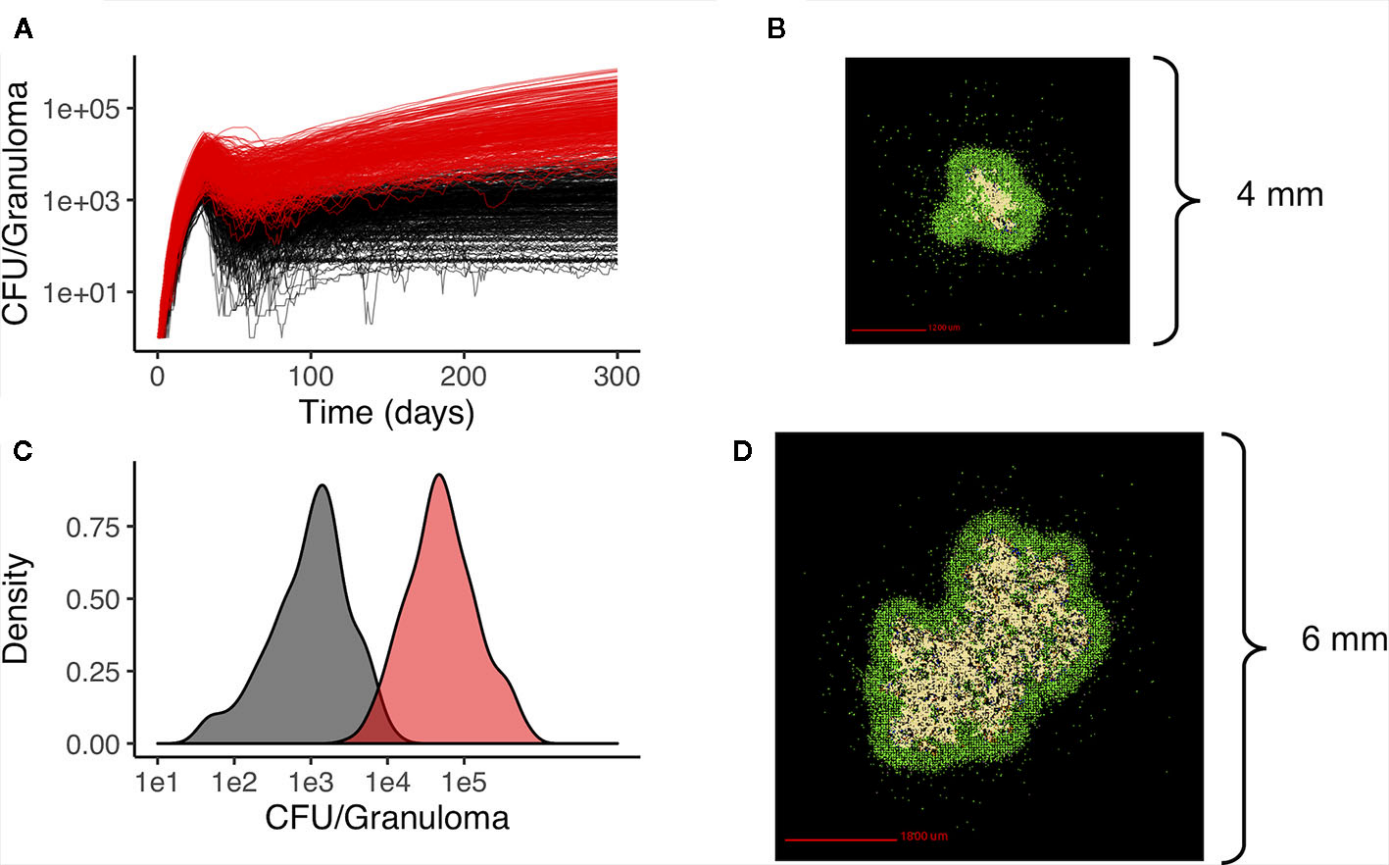
Heterogeneous granulomas generated using the computational model *GranSim*. There are two groups of in silico granulomas at day 300 post infection: low CFU granulomas (black/gray, n=354) and high CFU granulomas (red, n=352). High CFU granulomas have increasing CFU over time relative to the more stable lower CFU granulomas **(A)**. **(C)** shows the distribution of CFU per granuloma in the low CFU group (black) and the high CFU group (red) at day 300. **(B)** shows an example of a low CFU *in silico* granuloma and **(D)** shows an example of a high CFU granuloma. In both simulations the colors represent: macrophages green; resting; blue, active; orange, infected; red, chronically infected), T cells (IFN-gamma producing; pink, cytotoxic, purple; regulatory, light blue), and caseated regions (tan).

We generate two distinct libraries of granulomas that are heterogeneous in bacterial load and cellular composition: one categorized as low-CFU (colony-forming unit, equal to the number of bacteria in the simulation) granulomas and the other as high-CFU granulomas. The low-CFU granulomas are smaller in size and have CFU/granuloma that are more stable over time, whereas the high-CFU granulomas are larger in size, have increasing CFU over time, and have higher levels of caseum. To generate the low-CFU granulomas, we sampled 500 parameter sets based on ranges for host immune system parameters listed in **Table 1** using Latin Hypercube Sampling (LHS) (Pienaar et al., 2015a). Using the simulation outputs at day 300 for granuloma size and CFU, we performed sensitivity analysis using partial rank correlation coefficients to determine parameters that have the most significant impact on those two outcomes (Marino et al., 2008). A total of 400 high-CFU granulomas were generated by increasing or decreasing the upper and lower bounds of the parameter ranges that have the strongest correlation with granuloma size and CFU, as well as initializing simulations with multiple infection locations to generate larger granulomas. Parameter ranges for all granulomas are shown in **Table 1**. Low-CFU granulomas are simulated on a 200 by 200 compartment square grid representing a 4 by 4 mm section of lung tissue (each grid compartment has a side-length of 20 microns), whereas the high-CFU are run on 300 by 300 compartment grid representing 6 by 6 mm. Note that we simulate the small granulomas on a smaller grid for computational efficiency, as the larger is not required. At day 300, a total of 354 low-CFU granulomas and 352 high-CFU granulomas still had bacteria and were selected for treatment simulations. **Figure 1** shows CFU per granuloma of these two groups.

### Plasma Pharmacokinetic Model

**Figure 2:**
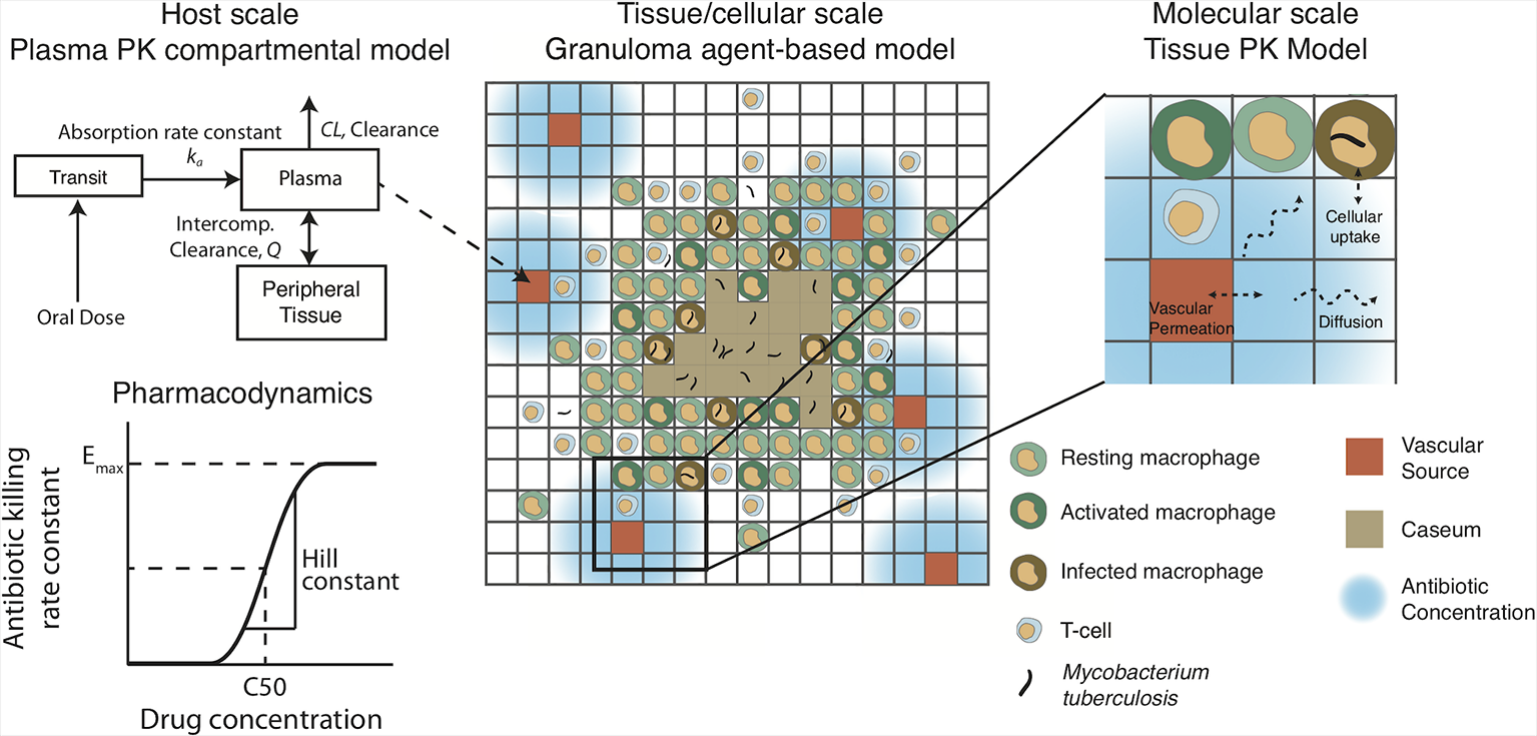
Pharmacokinetic/pharmacodynamic dynamics in *GranSim*. Plasma concentration is simulated with a two-compartment pharmacokinetic (PK) model with one [ethambutol (EMB) and pyrazinamide (PZA)] or two [isoniazid (INH) and rifampin (RIF)] transit compartments to capture oral absorption. The amount of drug added or subtracted through the vascular sources in the agent-based spatial grid depends on local gradients of antibiotics. Antibiotics on the grid can diffuse, degrade, bind to extracellular material (such as caseum) and partition into macrophages. Based on intra- or extracellular concentrations in each grid compartment, a killing rate constant based on a Hill curve determines the probability per time step that a given bacterium will die due to exposure to antibiotics.

The plasma PK model is comprised of a two-compartmental model with one or two transit compartments that simulate oral absorption. INH and RIF follow a two-absorption compartment model based on previously developed PK models, whereas EMB and PZA are simulated with one absorption compartment based on best fits and other PK models (Jonsson et al., 2011; Kjellsson et al., 2012; Denti et al., 2015; Pienaar et al., 2015a; Zimmerman et al., 2017). The two-compartment model simulates distribution between plasma and peripheral tissue, and antibiotics are eliminated with a first-order clearance rate constant (**Figure 2**). Pharmacokinetic variability can be introduced by varying the parameters of the plasma PK model based on reported variability in the parameters (**Table 2**).

**Table 2:** Plasma pharmacokinetic parameters listed with the ranges used to calibrate the tissue pharmacokinetic parameters, as well as the parameter values for the average and low pharmacokinetic (PK) exposure treatment groups.

Parameter	Units	Min	Max	Average PK	Low PK	Source
INH Absorption rate constant	1/h	0.50	6.0	3.25	0.57	Fit to data from (Prideaux et al., 2015)
INH Intercompartmental clearance rate constant	L/(h*kg)	0.20	0.70	0.45	0.67	Fit to data from (Prideaux et al., 2015)
INH Central compartment volume of distribution	L/kg	0.50	3.0	1.75	2.5	Fit to data from (Prideaux et al., 2015)
INH Peripheral compartment volume of distribution	L/kg	25	40	32.5	37	Fit to data from (Prideaux et al., 2015)
INH Plasma clearance rate constant	L/(h*kg)	0.0080	0.070	0.039	0.061	Fit to data from (Prideaux et al., 2015)
RIF Absorption rate constant	1/h	0.40	2.5	1.5	0.41	Fit to data from (Prideaux et al., 2015)
RIF Intercompartmental clearance rate constant	L/(h*kg)	2.0	5.9	3.9	3.95	Fit to data from (Prideaux et al., 2015)
RIF Central compartment volume of distribution	L/kg	0.18	0.57	0.38	0.48	Fit to data from (Prideaux et al., 2015)
RIF Peripheral compartment volume of distribution	L/kg	0.32	0.97	0.64	0.9	Fit to data from (Prideaux et al., 2015)
RIF Plasma clearance rate constant	L/(h*kg)	0.050	0.30	0.175	0.3	Fit to data from (Prideaux et al., 2015)
EMB Absorption rate constant	1/h	0.10	0.80	0.45	0.1	Fit to data from (Jonsson et al., 2011)
EMB Intercompartmental clearance rate constant	L/(h*kg)	0.45	0.70	0.57	0.56	Fit to data from (Jonsson et al., 2011)
EMB Central compartment volume of distribution	L/kg	0.80	1.95	1.37	1.7	Fit to data from (Jonsson et al., 2011)
EMB Peripheral compartment volume of distribution	L/kg	8.1	12.7	10.4	12.6	Fit to data from (Jonsson et al., 2011)
EMB Plasma clearance rate constant	L/(h*kg)	0.3	1.0	0.65	0.99	Fit to data from (Jonsson et al., 2011)
PZA Absorption rate constant	1/h	0.55	0.75	0.65	0.60	Fit to data from (Prideaux et al., 2015)
PZA Intercompartmental clearance rate constant	L/(h*kg)	0.10	0.70	0.40	0.35	Fit to data from (Prideaux et al., 2015)
PZA Central compartment volume of distribution	L/kg	0.25	0.75	0.50	0.74	Fit to data from (Prideaux et al., 2015)
PZA Peripheral compartment volume of distribution	L/kg	0.010	0.050	0.030	0.050	Fit to data from (Prideaux et al., 2015)
PZA Plasma clearance rate constant	L/(h*kg)	0.010	0.050	0.030	0.050	Fit to data from (Prideaux et al., 2015)

### Tissue Pharmacokinetic Model

The plasma PK model is linked to the agent-based environment through blood vessels placed on the simulation grid (**Figure 2**). Based on the difference between the plasma concentration and local tissue concentration in the compartments surrounding a blood vessel and the permeability of the antibiotic through blood vessel walls, a flux of antibiotic through the vessel wall is calculated as in previous work (Pienaar et al., 2015a). Antibiotics in the tissue (on the simulation grid) undergo a series of distribution events: diffusion, binding to extracellular material such as caseum, partitioning into macrophages, and degradation. Implementation of vascular permeation, diffusion, binding and degradation is as previously published (Cilfone et al., 2015; Pienaar et al., 2015a). Antibiotics on the simulation grid can be tracked as free molecules, bound to extracellular material, or partitioned into macrophages. When calibrating and fitting to data, we use total drug concentration in a grid compartment, but only free or intracellular antibiotic is used to determine antimicrobial activity, depending on the location of bacteria. Calibrated tissue PK parameters are given in **Table 3** (see below for calibration datasets).

**Table 3:** The calibrated tissue pharmacokinetic (PK) parameters for each antibiotic.

Parameter	INH	RIF	EMB	PZA	Source
Extracellular degradation rate constant (1/s)	6.94e-8	3.90e-8	1.73e-8	1.34e-8	Fit to data from (Prideaux et al., 2015; Zimmerman et al., 2017)
Intraceullar degradation rate constant (1/s)	2.84e-6	2.59e-4	8.75e-6	2.26e-3	Fit to data from (Prideaux et al., 2015; Zimmerman et al., 2017)
Effective diffusivity* (cm^2^/s)	6.58e-7	5.08e-8	5.20e-7	3.24e-6	Fit to data from (Prideaux et al., 2015; Zimmerman et al., 2017)
Cellular accumulation ratio	1.13	24	5.95	0.593	Fit to data from (Prideaux et al., 2015; Zimmerman et al., 2017)
Vascular permeability (cm/s)	1.34e-6	2.65e-7	1.33e-7	8.62e-6	Fit to data from (Prideaux et al., 2015; Zimmerman et al., 2017)
Permeability coefficient	0.25	3.3	7.4	1	Fit to data from (Prideaux et al., 2015; Zimmerman et al., 2017)
Fraction unbound to caseum	1	0.052	0.35	1	Fit to data from (Prideaux et al., 2015; Sarathy et al., 2016; Zimmerman et al., 2017)

*Guided by estimates from (Pruijn et al., 2008)

### Sequential Pharmacokinetic Model Calibration Scheme

**Figure 3:**
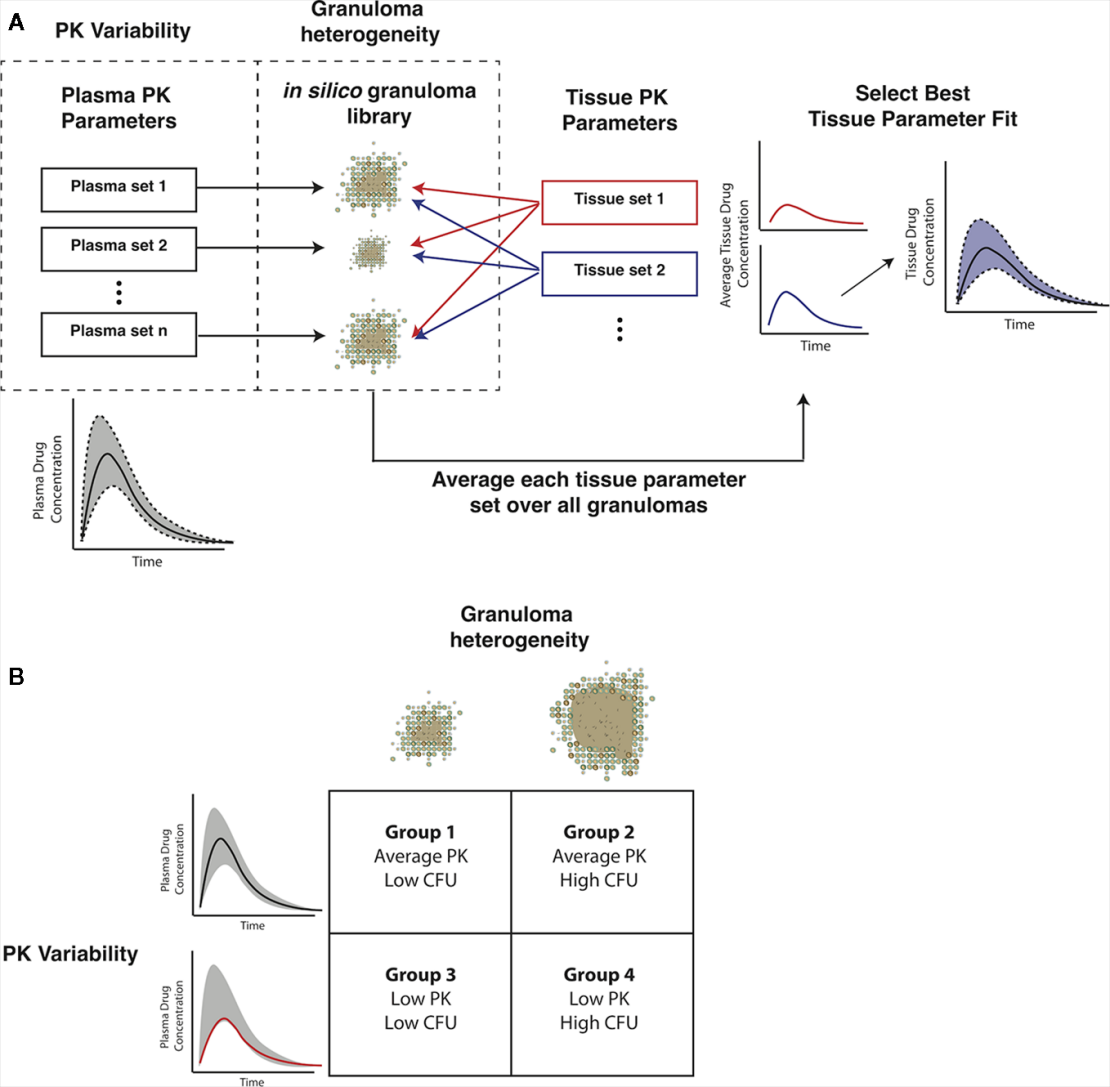
Capturing pharmacokinetic variability and granuloma heterogeneity in pharmacokinetic (PK) calibration and treatment simulations. **(A)** shows our strategy. Based on population variability and ranges in plasma PK parameters, sets of plasma PK parameters are sampled and assigned to a set of *in silico* granulomas. Based on experimentally guided ranges for tissue PK parameters, a set of tissue PK parameters is obtained using Latin Hypercube Sampling (LHS). Simulations then predict antibiotic concentrations in the tissue. The average concentration over all granulomas for a given tissue PK parameter set is calculated and compared to experimental lesion concentrations. **(B)** shows the four types of treatment simulations that capture biologically relevant PK variability and granuloma heterogeneity: average PK exposure with low or high CFU granulomas and low PK exposure with low or high CFU granulomas.

Gradients between plasma and tissue concentrations drive the amount of antibiotic delivered into the agent-based model simulation through blood vessels, so fitting tissue PK parameters to match the experimentally observed spatial distribution and average antibiotic concentrations in granuloma lesions requires incorporating both plasma PK variability and granuloma heterogeneity. We have devised a pipeline for incorporating these factors into *GranSim* (**Figure 3A**). Using our *in silico* granuloma library, each granuloma is assigned a different plasma PK set sampled using LHS from parameter ranges that capture biological variability (**Table 2**). Next, changes in antibiotic tissue concentrations over time are simulated for each granuloma with 200 tissue PK parameter sets sampled using LHS. For each tissue PK parameter set, the results from each granuloma are averaged at each time point, and then compared to experimental data. The tissue PK parameter set that both minimizes the sum of the squared error between average granuloma concentration and the experimental lesion concentrations, as well as provides a good visual fit to the data is chosen as the calibrated tissue PK parameter set. Tissue PK parameter ranges for calibration sampling are listed in **Table 3**.

### Pharmacokinetic Data

Plasma and tissue PK parameters for INH, RIF, and PZA are calibrated using plasma and lesion antibiotic concentrations measured in resected lung samples from patients with drug-refractory TB (Prideaux et al., 2015). EMB concentrations in rabbit TB granulomas are used to calibrate tissue PK parameters, based on rabbit plasma PK parameters (Zimmerman et al., 2017). Since tissue PK parameters are based on physical properties and interactions between drug molecules and tissue, we assume that tissue PK parameters in rabbits and humans are similar. To simulate human treatment with EMB, we replace the rabbit plasma PK parameters with human parameters fit to population PK measurements (Jonsson et al., 2011).

### Pharmacodynamic Model

The pharmacodynamic model involves evaluating a concentration-dependent killing rate constant derived from a Hill curve:

$$k=E_{max}\frac{C^{h}}{C^{h}+C_{50}^{h}}$$

The killing rate constant *k* (units of 1/timestep or 1/10 min) is dependent on the variable concentration (*C*), the maximum killing rate constant (*E_max_*), the concentration at half maximal killing (*C_50_*), and the Hill curve constant (*h*). The concentration used to determine the antibiotic killing rate constant is based only on free drug concentration. Parameters *E_max_*, *C_50_*, and *h* need to be determined for each antibiotic and for each bacterial subpopulation (replicating extracellular, non-replicating extracellular, and intracellular). To fit these parameters, we use *in vitro* dose-response assays from individual experiments from the literature of Mtb growth/death under varying antibiotic concentrations (see **Table 4** for parameters and references for data and refer to **Supplementary Figure 1** for calibrated fits to dose-response curves). In the present model, we do not include drug-drug interactions, so the highest single antibiotic killing rate constant for each antibiotic within a specific grid compartment in the simulation is used as the effective antibiotic killing rate constant for that location (Bhusal et al., 2005; Pienaar et al., 2015b). This assumption isolates the impact of PK variability and granuloma heterogeneity on granuloma sterilization within this study.

**Table 4:** Pharmacodynamic parameter and sources for data used for parameter fitting/estimation. Units of 1/timestep represent per model timestep of 10 min.

Parameter	INH	RIF	EMB	PZA	Sources
Intracellular C_50_ (mg/L)	0.070	20	5.22	70	(Jayaram et al., 2003; Jayaram et al., 2004; Hartkoorn et al., 2007; Sarathy et al., 2018)
Extracellular, replicating C_50_ (mg/L)	0.015	1.23	0.05	370	(Jayaram et al., 2003; Jayaram et al., 2004; Hartkoorn et al., 2007; Sarathy et al., 2018)
Extracellular, Non-replicating C_50_ (mg/L)	17.7	81	1000	370	(Lakshminarayana et al., 2015; Sarathy et al., 2018)
Intracellular Emax (1/timestep)	0.0056	0.014	0.026	0.0006	(Jayaram et al., 2003; Jayaram et al., 2004; Hartkoorn et al., 2007; Sarathy et al., 2018)
Extracellular Emax (1/timestep)	0.0056	0.019	0.025	0.007	(Jayaram et al., 2003; Jayaram et al., 2004; Hartkoorn et al., 2007; Sarathy et al., 2018)
Intracellular hill constant, h	1	0.5	2.5	3.2	(Jayaram et al., 2003; Jayaram et al., 2004; Hartkoorn et al., 2007; Sarathy et al., 2018)
Extracellular hill constant, h	1	0.5	1.5	1	(Jayaram et al., 2003; Jayaram et al., 2004; Hartkoorn et al., 2007; Sarathy et al., 2018)

### *In Silico* Antibiotic Treatment of Granulomas

Treatment simulations are executed by choosing the non-sterile set of low and high CFU *in silico* granulomas that have formed 300 days post infection (in the absence of antibiotics). The doses for the standard regimen are based on CDC recommended adult doses for each of the four antibiotics: INH, 5 mg/kg; RIF, 10 mg/kg; EMB, 17 mg/kg; PZA 21 mg/kg (Nahid et al., 2016). Simulated treatments use daily doses of each antibiotic. Treatment simulations are administered for a maximum of 180 days, which are based on standard regimen length (Nahid et al., 2016); simulations are stopped once granulomas sterilize to reduce computational resource use. After treatment, we calculate a simulated early bactericidal activity (EBA), which is defined as the rate of decrease of log_10_ (CFU) per day. For example, the EBA for 0–2 days is calculated as (log_10_(CFU day 0)-log_10_(CFU day 2))/2. We simulate four groups of granulomas to incorporate PK variability and granuloma heterogeneity (**Figure 3B**). Group 1 has population average plasma PK exposure (AUC) with low-CFU granulomas and Group 2 has average PK exposure with high-CFU granulomas. Groups 3 and 4 both have low plasma PK exposure, with low and high-CFU granulomas respectively. Plasma PK parameter values for the low and average PK exposure are listed in **Table 2**.

## Results

### Pharmacokinetic Model Captures Plasma and Lesion Variability in Antibiotic Concentrations

Heterogeneity in antibiotic exposure within granulomas is the result of two factors. Differences in plasma drug concentrations among individuals can be due to differences in drug absorption and elimination rates, and these are reflected in distributions of plasma PK parameters across a population. Additionally, granuloma structural heterogeneity (including differences in size and composition) can lead to differences in antibiotic exposure at the lesion level. To capture both sources of heterogeneity, which occur at different length scales, we devised a sequential calibration scheme to calibrate the PK model from data (**Figure 3**; parameters in **Tables 2** and **3**).

**Figure 4:**
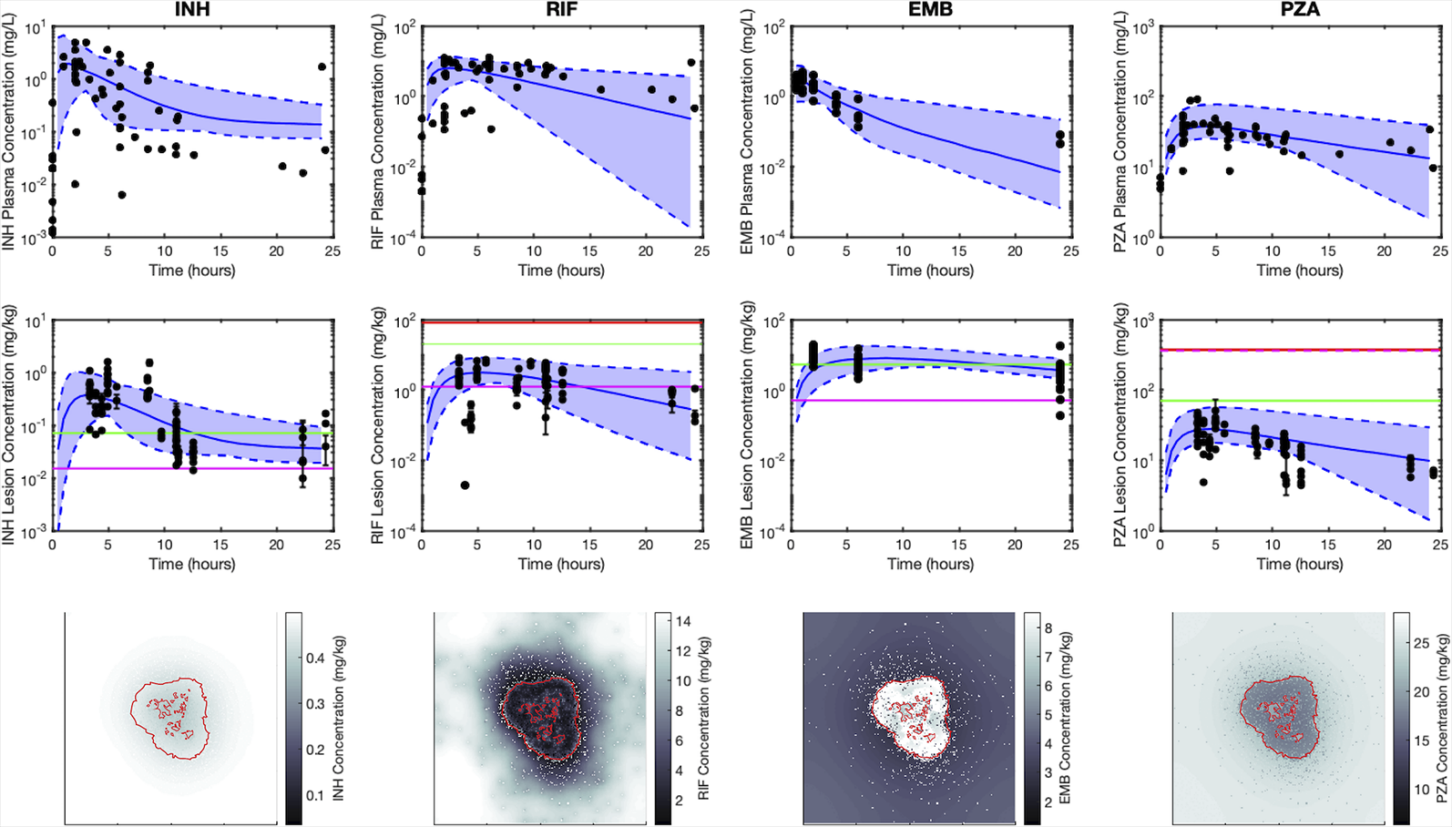
Simulations capture both the experimentally observed temporal and spatial antibiotic concentrations. Simulations and data for each antibiotic [isoniazid (INH), rifampin (RIF), ethambutol (EMB), and pyrazinamide (PZA)], dosed singly, are shown in different columns, respectively. The top row shows plasma concentrations and the middle row shows average lesion concentrations with varying plasma pharmacokinetic (PK) parameters (median, solid blue line; range between minimum and maximum of simulations, blue shade) and experimentally measured antibiotic concentrations (black points). Concentrations in granulomas are in mg/kg (assuming tissue density is approximately 1 kg/L), and reflect the sum of concentrations of free, bound and intracellular drug. Horizontal lines represent the C_50_ values for intracellular (green), extracellular replicating (magenta) and non-replicating (red) subpopulations of Mtb (C_50_ values not shown are above the range of lesion concentrations displayed on the plot). Data in the middle row are measurements from human granulomas (INH, RIF, and PZA (Prideaux et al., 2015)) and rabbit granulomas [EMB (Zimmerman et al., 2017)]. The bottom row shows spatial distribution of antibiotics in *GranSim* at the time of the maximal average lesion concentration. Red outlines indicate edge of granuloma (outer line) and caseated locations (inner lines).

**Figure 4** shows antibiotic total concentrations (sum of free and bound) within both plasma and granulomas for all four first-line antibiotics (INH, RIF, EMB, and PZA). Results are shown for 24 h following an oral dose and compared to experimental data. Using our sequential calibration scheme, we capture a large proportion of the experimentally observed antibiotic concentration data in both plasma and granulomas. The C_50_ values for each bacterial subpopulation, obtained by fitting the data referenced in **Table 3**, indicate the free-drug concentration when a given antibiotic is at half its maximum bacterial killing rate. INH, RIF, and EMB achieve sufficient concentrations to kill extracellular replicating Mtb for a majority of the dosing period. Both INH and EMB can achieve concentrations above the C_50_ for intracellular Mtb. Only RIF approaches concentrations necessary to achieve bactericidal activity against non-replicating Mtb. Based on average granuloma concentrations, PZA appears to have little sterilizing activity in granulomas.

**Figure 5:**
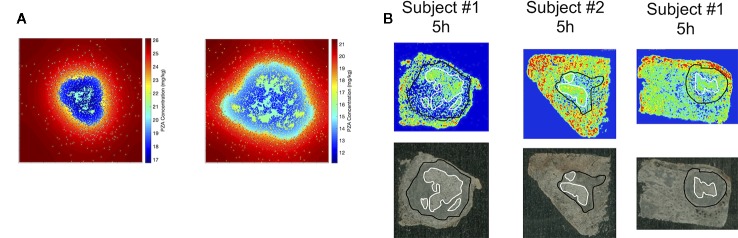
Comparison of spatial distribution of pyrazinamide (PZA) in GranSim **(A)** and in experimental images of granulomas using matrix-assisted laser desorption/ionization mass spectrometry imaging (MALDI-MSI) **(B)**. The simulation images show heat maps of the spatial distribution of PZA at 5 h after a single-PZA dose. In the simulated concentration heat maps, shown in color to mimic the images from MALDI-MSI **(A)**, the red area corresponds to lung tissue outside of the granuloma, the darker blue regions indicates regions inside the granuloma with higher densities of macrophages, and the lighter blue to green sections show correspond to caseated regions. Both simulation images are on a 200 by 200 grid, representing a 4 mm by 4 mm section of lung tissue. Experimental images **(B)** show PZA distribution in granulomas imaged with MALDI-MSI, with granuloma boundary outlined in black, and caseated regions outlined in white. Both simulation and experiments show some accumulation of PZA inside caseous regions, relative to the cellular portions of the granuloma.

**Figure 4** also shows antibiotic total concentrations as a function of position throughout the grid, at the time of maximal average granuloma drug concentration following a single oral dose of each antibiotic in the same *in silico* granuloma, shown in grayscale to allow for the illustration of gradual concentration changes. INH shows a relatively homogenous distribution in the lesion that rapidly clears as INH is eliminated in the plasma. RIF accumulates poorly in granulomas at early time points but can slowly accumulate in the caseum following multiple doses (**Supplementary Figure 2**). EMB tends to accumulate in regions with a high density of macrophages but fails to diffuse into caseum significantly. PZA shows a slight accumulation in caseum relative to the macrophage-rich regions of the granuloma. To further validate our model, **Figure 5** shows PZA distribution identified experimentally using matrix-assisted laser desorption/ionization mass spectrometry imaging (MALDI-MSI) as we have done previously (Prideaux et al., 2015) and compares the PZA signal intensity distribution to two simulated granulomas. Overall, our simulated distributions for other antibiotics agree with observations made through MALDI-MSI in TB granulomas (Prideaux et al., 2015; Zimmerman et al., 2017). These qualitative features observed in the simulations for each antibiotic were not used in calibrating the tissue PK parameters, but rather resulted from estimating and fitting the tissue PK parameters to average granulomas concentrations (**Table 1**).

### Single-Drug Treatments Sterilize Granulomas at Different Rates and to Different Extents

**Figure 6:**
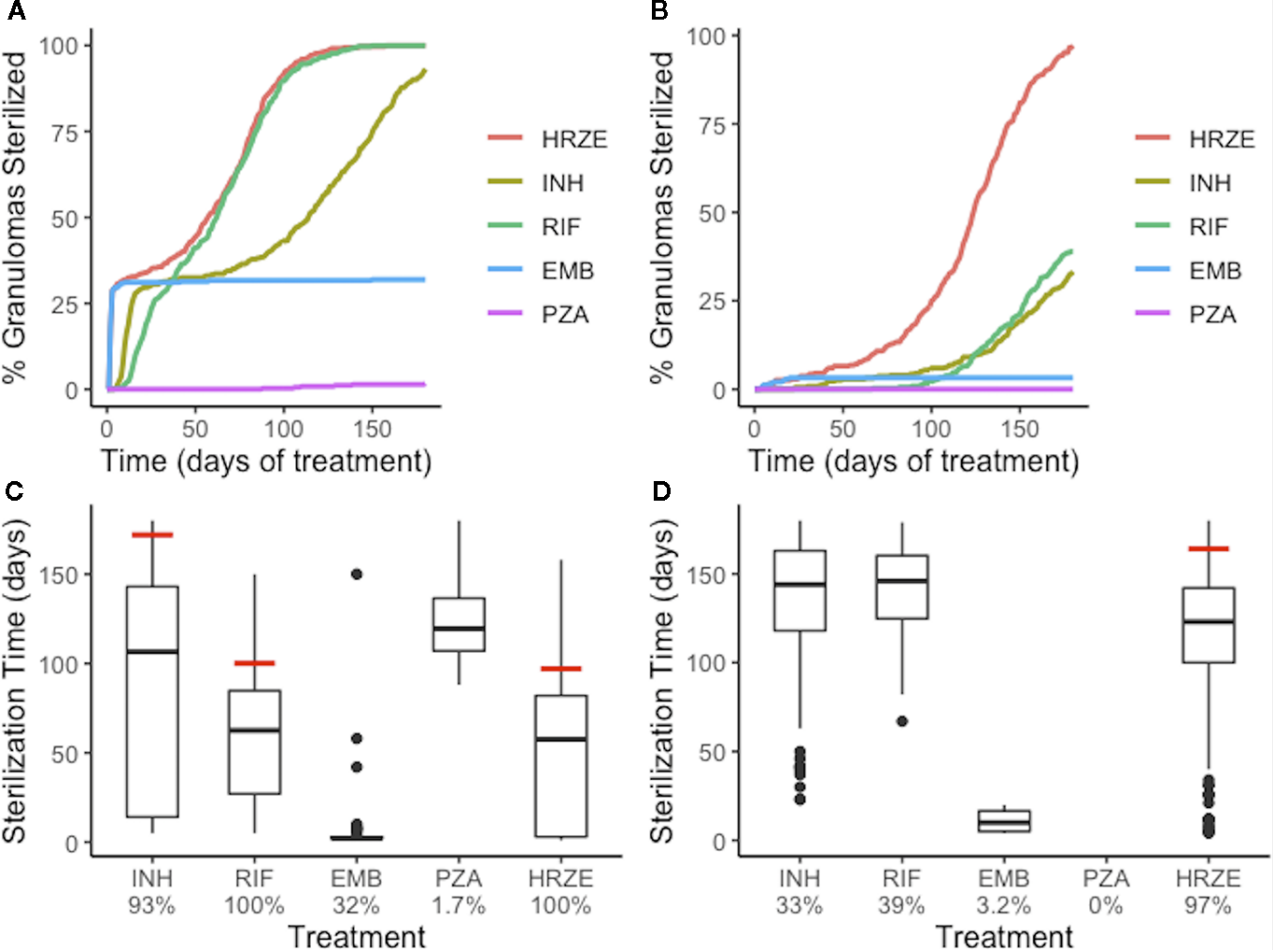
Single-antibiotic treatments and combination therapy of low-CFU **(A, C)** and high-CFU **(B, D)** granulomas show different sterilizing rates and extents for each of the first-line antibiotics and all four antibiotics together (HRZE). **(A, B)** show the percentage of granulomas sterilized over the course of treatment for both groups of granulomas. **(C, D)** show the distribution of sterilization times for only the granulomas that sterilized for each treatment, with the time when 90% of granulomas were sterilized indicated by a red line. Percentage below each treatment indicates the total percentage of granulomas that sterilized. For example, EMB sterilized 32% of low-CFU granulomas **(C)**, and of those sterilized granulomas, a majority of them sterilized in the first few days (indicated by the box plot collapsing to a line).

We next tested the abilities of each first-line antibiotic, when dosed alone, to sterilize granulomas with average plasma PK exposure and low or high-CFU (Groups 1 and 2 of **Figure 3**). The rates and extents of sterilization differ for each antibiotic in low-CFU granulomas, as shown in **Figure 6A**, due to differences in sterilizing activity against various subpopulations of bacteria as well as the antibiotic distribution within granulomas. After 180 days of treatment, single-drug therapy with RIF sterilizes all low-CFU granulomas. INH sterilized 93% low-CFU granulomas. EMB and PZA each sterilize just 32% and 1.7% of low-CFU granulomas, respectively. INH and EMB all have early sterilizing ability and were able to sterilize 29% and 31% of granulomas after two weeks, respectively. RIF alone only sterilized 5% by 2 weeks, and PZA failed to sterilize any granulomas by two weeks.

Our simulations show that INH can quickly distribute within granulomas, and in sufficient concentrations to kill both intracellular and extracellular replicating bacteria, and therefore provides rapid sterilization for some granulomas, as shown in **Figures 4** and **5**. However, with poor sterilizing ability against non-replicating Mtb found in caseum (Sarathy et al., 2018), INH usually requires many months to sterilize granulomas that have a high number of non-replicating Mtb and leads to the drawn-out sterilization of non-replicating Mtb in INH-treated granulomas. EMB, similar to INH, has poor ability to kill non-replicating Mtb, so our simulations show it is only able to sterilize a subset of granulomas, even though it distributes throughout cellular regions of the granuloma. However, it does rapidly kill both extracellular replicating and intracellular Mtb, indicated by the percentage of granulomas sterilized by two weeks, which is consistent with favorable early bactericidal activity (EBA) for EMB (Donald and Diacon, 2008). Because INH and EMB are bacteriostatic and have low ability to kill non-replicating Mtb, sterilization time (for INH) and total Mtb remaining in the granuloma (for EMB) are highly correlated with the initial number of non-replicating bacteria present in the granuloma (**Supplementary Figure 3**). RIF shows more complete sterilization of low-CFU granulomas than any other individual antibiotic as it has some sterilizing ability against each subpopulation of bacteria.

We observe similar trends with single-drug treatments in high-CFU granulomas (**Figure 6B**). Overall, the sterilization times are longer when compared to low-CFU granulomas. INH and RIF both sterilize lower percentages of the high-CFU granulomas than they do low-CFU granulomas. High-CFU granulomas are also more likely to have higher total numbers of non-replicating Mtb, decreasing the ability of INH to completely sterilize these granulomas. RIF, with weakened ability to kill intracellular Mtb due to low granuloma concentrations, fails to kill all intracellular Mtb in some granulomas. This weakness is amplified in larger granulomas, slowing diffusion of antibiotics into the granulomas.

### Specialization of Individual Antibiotics Contributes to Success of Combination Therapy

Combination therapy—all four first-line antibiotics—sterilizes low-CFU granulomas at nearly the same rate as the best single-antibiotic treatment (RIF) (**Figure 6**). All granulomas are sterilized after 147 days of combination therapy, with 33% sterilized after 2 weeks. The difference in early versus late sterilizing ability for the single-drug treatments is one reason why the combination therapy shows faster and more complete sterilization than any one drug on its own. Early in treatment, INH and EMB do much of the killing, and the presence of RIF completes the sterilization.

The benefit of combination therapy is more dramatic for high-CFU granulomas (Group 2 of **Figure 3**). Here, treatment with INH or RIF show only 33% and 39% sterilization after 180 days of therapy, respectively, compared to 97% of granulomas sterilized with HRZE (**Figure 6D**). Although RIF is able to sterilize granulomas as well as HRZE in low-CFU granulomas, the same behavior is not observed in high-CFU granulomas. RIF is relatively slow at killing intracellular bacteria. In the low-CFU granulomas, the number of intracellular Mtb is low enough where RIF can kill these bacteria eventually. In high-CFU granulomas, RIF is not always able to kill intracellular Mtb fast enough to keep up with its replication, and therefore fails to sterilize all high-CFU granulomas. The presence of INH and EMB provide assistance in killing the intracellular Mtb, so the combination of antibiotics allows for more complete sterilization. Our model predicts that the different abilities to kill each of the subpopulations of bacteria and the different distributions within granulomas complement each other in combination therapy.

During combination therapy (HRZE), a majority of bacterial death is due to antibiotics; antibiotics are responsible for roughly an order of magnitude more bacterial death than the immune response, and two orders more than bacterial death in caseum representing a lack of oxygen and nutrients (**Supplementary Figure 4**). This trend is consistent across the single-drug treatments with the exception of PZA, which shows the poorest efficacy and thus allows for continued bacteria growth and continued slow killing *via* the immune response.

### High-CFU and Low PK Exposure Lengthen Sterilization Times During Combination Therapy

**Figure 7:**
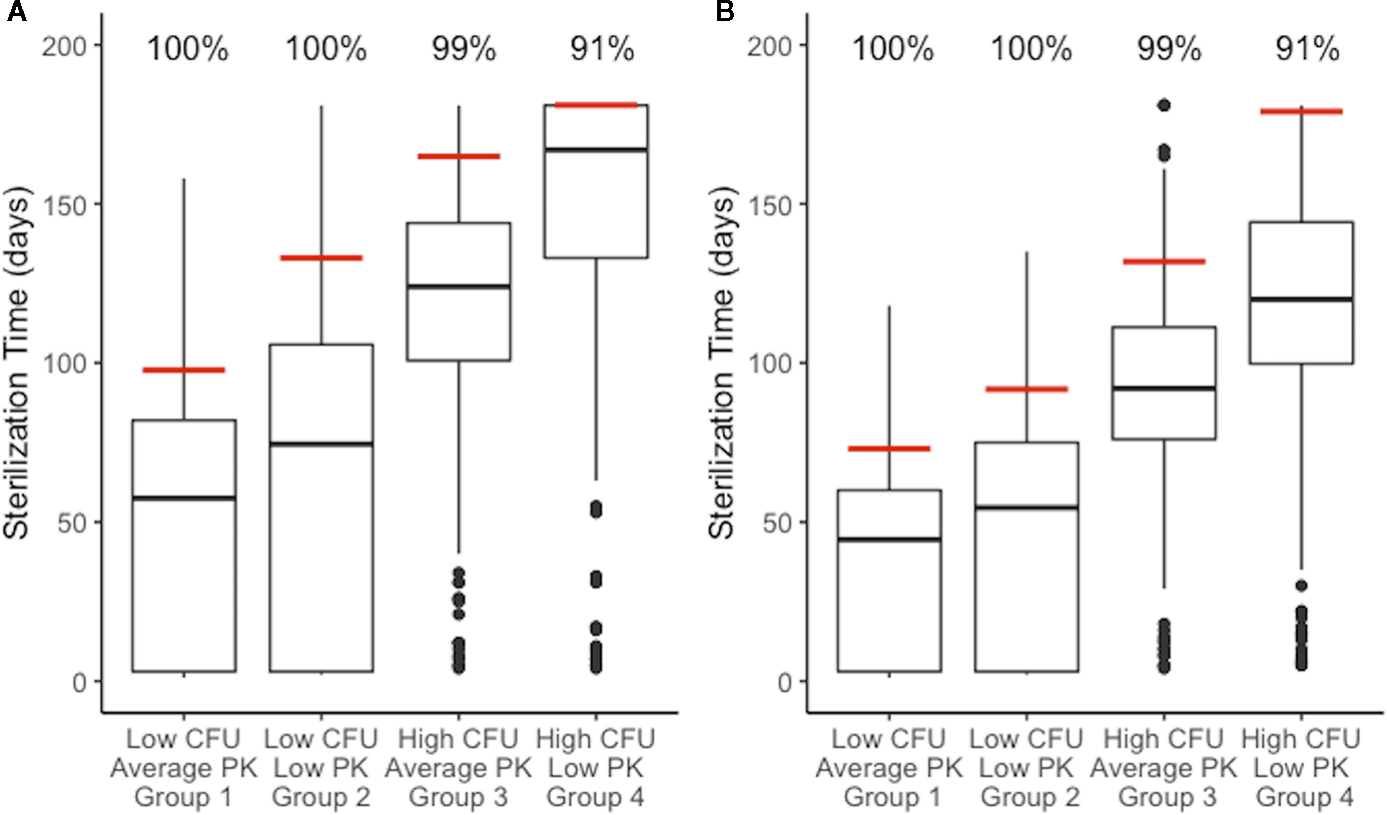
Distributions of sterilization times for different granuloma treatment groups, referenced in **Figure 3B**, treated with HRZE indicate factors that negatively impact sterilization. **(A)** shows simulations of the standard regimen (HRZE). **(B)** shows the simulations of high rifampin (RIF) dose treatments (20 mg/kg). Each boxplot shows the sterilization time distribution of a treatment group, with outlying simulations as dots and the red line indicating the time of 99% sterilization.. Low CFU granulomas with average pharmacokinetic (PK) exposure sterilize the fastest. Low CFU granulomas with low PK exposure show a shift to longer sterilization times compared with average exposure. Similarly, high CFU granulomas with average exposure sterilize faster than high CFU granulomas with low exposure. Results for low CFU and high CFU with average PK are shown in **Figure 6** and are plotted again here for comparison.

We next tested how sterilization time and thus the necessary length of treatment is affected by plasma PK variability between individuals and granuloma heterogeneity. We compared the sterilization times of all four granuloma groups (**Figure 3B**) when treated with daily doses of HRZE. **Figure 7A** shows the distribution of sterilization times for each of these treatment scenarios. Simulating the low-CFU granulomas with low PK exposure (Group 2) results in a shift in the distribution towards longer sterilization times relative to average PK exposure (Group 1), with the 90% sterilization time increasing from 97 to 133 days. In contrast, 165 days of HRZE are required to sterilize 90% of the high-CFU granulomas with average PK exposure, and 90% sterilization cannot be reached within 6 months of treatment when those same granulomas have low PK exposure.

With some granulomas failing to sterilize after 180 days of treatment, we sought to analyze the characteristics of those granulomas. We grouped our granulomas into four different “risk” categories: low (sterilize in under 90 days of HRZE), medium (sterilize between 90 and 150 days), high (sterilize after 150 days), and unsterilized. For each of these groups, we compared characteristics of the granulomas before treatment to see what types of granulomas have different levels of risk. Unsterilized and high-risk granulomas tend to be higher in CFU, size, and amount of caseation (**Supplementary Figure 5**), with median CFU/granuloma levels before treatment of 1.1x10^5^, 6.0x10^4^, 2.1x10^4^, and 1x10^3^ for the unsterilized, high, medium, and low risk categories. However, these pretreatment characteristics are not sufficient in predicting whether a specific granuloma will fail to sterilize during treatment, as there are some granulomas with high CFU, diameter, and caseation that sterilize within 90 days. Although these low risk granulomas look like high risk or unsterilized granulomas, they have higher percentages of intracellular Mtb. At the beginning of treatment with HRZE, these intracellular bacteria can be quickly killed, making the granulomas easier to sterilize.

**Figure 8:**
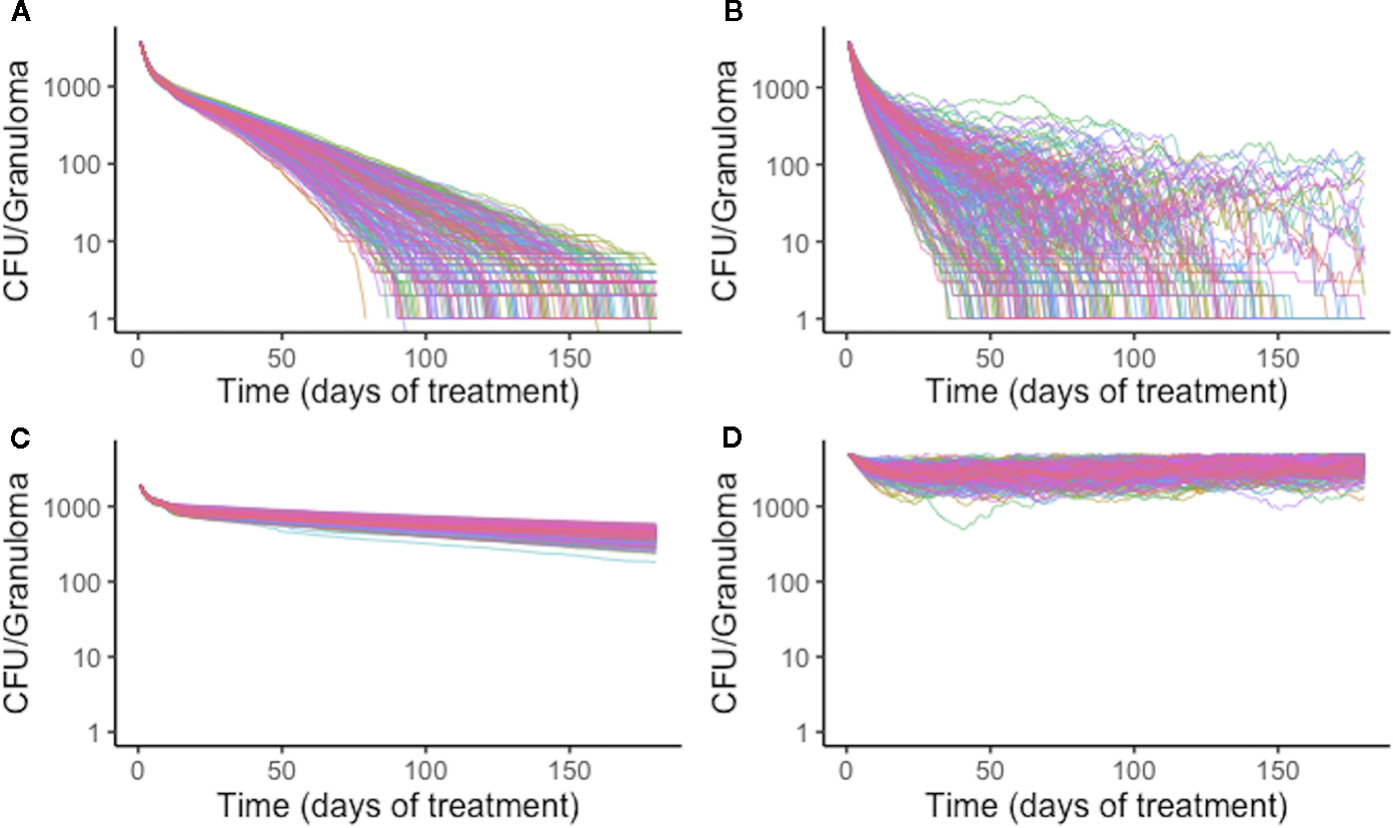
Simulation treatment outcomes of single-drug treatments of the same *in silico* granuloma vary with different plasma pharmacokinetic (PK) parameter sets. A single granuloma was treated with each of the single-drug treatments with 200 different plasma PK parameter sets. Above shows the CFU for each granuloma simulation over time during treatment for isoniazid (INH) **(A)**, rifampin (RIF) **(B)**, ethambutol (EMB) **(C)**, and pyrazinamide (PZA) **(D)**. The standard deviation of sterilization times for different plasma PK parameter sets for RIF normalized to mean sterilization time is 0.40. This indicates greater variability in sterilization times due to changes in plasma PK for RIF compared to INH, for which the value is 0.19. EMB and PZA have standard deviations of log-transformed CFU normalized to the mean at the end of treatment standard deviations of 0.033 and 0.034, respectively.

Variation in plasma PK exposure may impact treatment with some antibiotics more profoundly than others. To test this, we sampled a set of 200 plasma PK parameters from the ranges used in calibrating the PK model (**Table 2**). With each of these plasma PK parameter sets that generate different levels of exposure in plasma, we treated the same granuloma with each single-drug treatment (**Figure 8**). Overall, RIF is most impacted by natural variability in plasma exposure, and varying plasma PK parameters for RIF results in a wider spread of treatment outcomes than other antibiotics, ranging from a minimum sterilization time of 38 days to unsterilized granulomas by the end of treatment (**Figure 8**). This indicates that optimizing dose for RIF and other antibiotics that are particularly sensitive to variations in PK existing in human populations may be critical in designing better regimens.

### Treatment Time Can Be Shortened for Some Granulomas by Increasing the Dose of RIF

There have been numerous efforts to shorten TB treatment regimens and clinical trials that involve replacing one or more antibiotics in the standard regimen or increasing doses of the first-line antibiotics (Gillespie et al., 2014; Jindani et al., 2014). Increasing RIF dosage to 20 mg/kg is a strategy applied in several clinical trials (Diacon et al., 2007; Boeree et al., 2017; Peloquin et al., 2017), and is rational because it could lessen the impact PK variability has on RIF given our results (**Figure 7**). We investigated how increasing the RIF dose impacts granuloma sterilization time while accounting for granuloma heterogeneity and PK variability. To simulate high RIF dose treatments, we simulated each treatment group of granulomas with the same combination regimen as before but increased the RIF dose to 20 mg/kg.

Increasing the RIF dose in combination therapy results in shorter average sterilization times as compared to the standard combination therapy (**Figure 7B**). The 90% sterilization times for low-CFU granulomas decrease by 25 days for average PK and 41 days for low PK exposure. High-CFU granulomas with average PK exposure showed a decrease in 90% sterilization times by 33 days when treated with a high RIF dose, and the low PK exposure simulations increased the percent of sterilized granulomas from 62% to 91% (the latter giving a 90% sterilization time of 179 days). Overall, the improvement observed is greater in the low PK exposure simulations than in average PK simulations.

## Discussion

Treatment of drug-susceptible TB requires multiple months of antibiotics, after which treatment may still fail due to unsterilized granulomas. A better understanding of the first-line combination therapy, HRZE, will help to develop rational approaches to reduce treatment duration and improve cure rates. To analyze the use of first-line antibiotics and the factors that impact granuloma sterilization and conditions of treatment failure, we developed a computational framework that captures both granuloma heterogeneity and PK variability observed in human studies to determine the rate and extent of sterilization during treatment with first-line TB antibiotics at the granuloma scale.

To place the findings of our work into better context with clinical evidence that has been gathered on first-line TB antibiotics, we compared our simulations for single-drug treatments and combination treatments to early bactericidal activities (EBA) measured in multiple studies (**Table 5**). The EBA estimates based on the simulations are shown as the decrease in log_10_(CFU)/day for each treatment. Many of our simulated estimates are near the clinically measured EBA values (given as reported ranges or confidence intervals). For simulated EBA estimates that do not match clinical results, our simulations tend to predict lower EBA values than those observed clinically. Our EBA estimates account for the entire granuloma's CFU count, and it is possible that we predict lower EBAs as our simulations detect more remaining bacteria than those that would be detected clinically in sputum due to limitations of detection in assays used.

**Table 5:** Comparison of antibiotic treatment simulations to clinical early bactericidal activity (EBA) data. Table shows the simulation EBA, calculated as the decrease in log_10_ (CFU) per day over the day intervals indicted. Values reported are the mean daily decrease in CFU over all granulomas simulated with the standard regimen doses and average PK. Standard deviation is indicated in parenthesis. The clinical EBA values reported are taken from a number of studies and reviews. The simulation EBA for (0-*x*) days is calculated as (log_10_(CFU day 0)-log_10_)(CFU day x))/x.

	Simulation, Mean (SD)			Clinical		
Antibiotic	EBA 0-2 Days	EBA 0-5 Days	EBA 0-14 Days	EBA 0–2 Days	EBA 0–5 Days	EBA 0–14 Days
INH	0.16 (0.062)	0.13 (0.066)	0.079 (0.051)	Ranges from 0.37–0.77 involving 13 studies summarized in (Donald and Diacon, 2008)	0.25 (range of 0.19–0.40) as summarized in (Donald and Diacon, 2008)	Ranges from 0.189–0.192 involving two studies summarized in (Donald and Diacon, 2008)
RIF	0.15 (0.044)	0.12 (0.037)	0.086 (0.027)	Ranges 0.174–0.631 involving 8 studies summarized in (Donald and Diacon, 2008)	0.226 (SD 0.144) reported in (Sirgel et al., 2005)	^b^0.11 (SD 0.096) reported in (Donald and Diacon, 2008) from (Jindani et al., 1980)
EMB	0.45 (0.36)	0.20 (0.16)	0.082 (0.061)	0.25 (95% CI: 0.06–0.45) pooled in (Bonnett et al., 2017)	NA	^b^0.16 (SD 0.090) reported in (Jindani et al., 2003)
PZA	0.014 (0.009)	0.014 (0.007)	0.012 (0.006)	0.01 (95% CI: -0.07–0.09) pooled in (Bonnett et al., 2017)	NA	^b^0.11 (SD 0.038) reported in (Jindani et al., 2003)
HRZE	0.49 (0.34)	0.24 (0.15)	0.11 (0.052)	0.3 (95% CI: 0.09–0.50) pooled in (Bonnett et al., 2017)	^a^0.16 (95% CI: 0.09–0.24) pooled in (Bonnett et al., 2017)	0.16 (95% CI: 0.11–0.21) pooled in (Bonnett et al., 2017)

a EBA 0–7 Days

b EBA 2–14 Days

We found that typical PK variability and granuloma heterogeneity can create scenarios that profoundly impact sterilization rates and treatment success. The level of antibiotic concentration in plasma leads to commensurate concentrations within granulomas, creating differences in sterilization rates. Individuals with lower plasma PK exposure are at higher risk of antibiotic underexposure in selected granulomas. When coupled with complex and caseous granuloma structure with impaired vascular supply, this can lead to longer sterilization times using standard HRZE TB therapy (**Figure 7**). Other models using various experimental data, including hollow fiber experiments, show that low drug exposure can lead to decreased rates in bacterial killing (Srivastava and Gumbo, 2011), and have used variability in PK to predict variability in required treatment durations (Magombedze et al., 2018). The model we present builds on these findings by providing the ability to simulate sterilization in a granuloma, while accounting for human-based PK variability and granuloma structure. The benefit of simulating treatment in the context of the whole granuloma is that it includes the spatial microenvironments that can influence both antibiotic distribution and bacterial susceptibility or tolerance to antibiotics. Treating each Mtb as an individual agent also provides the ability to simulate treatment while accounting specifically for antibiotic resistance (Pienaar et al., 2018). Our model is a tool that can provide quantitative predictions and sterilization times for a given regimen at a granuloma level, the possibility to predict entire host treatment through linking of plasma pharmacokinetics, and the potential to search for optimal treatment regimens (Cicchese et al., 2017).

We show that treatment with any of the current first-line TB antibiotics alone is not sufficient to sterilize all granulomas, and that combinations of antibiotics result in more rapid and complete sterilization. Although RIF shows the best sterilizing ability on its own and is about as effective as HRZE in low-CFU granulomas, RIF alone fails to sterilize many of the high-CFU granulomas, where it only sterilizes 39% of granulomas compared to 97% with HRZE. Although our simulations predict that PZA sterilizes very few granulomas on its own, evidence suggests that PZA does show sterilizing ability when administered on its own, and suggests that our simulations underestimate its activity and that there is discrepancy between the *in vitro* activity of PZA and *in vivo* efficacy that our model does not capture (Irwin et al., 2016; Lanoix et al., 2016; Blanc et al., 2018).

Granulomas with increased CFU and lower antibiotic exposure can dramatically increase sterilization time and increase the risk of granulomas that do not completely sterilize. Granulomas with high risk of not sterilizing tend to be larger and have more CFU; however, the type of bacteria present in those granulomas may affect the risk of treatment failure as well. Granulomas with high CFU may still have a low risk of treatment failure if they have high percentages of intracellular Mtb. Because some of the antibiotics in HRZE are good at quickly killing this subpopulation, these granulomas that look like high risk granulomas pretreatment, quickly become low risk granulomas as treatment begins.

RIF is the antibiotic that provides the best sterilizing ability on its own, but also is the antibiotic that shows the highest inter-individual PK variability (Stott et al., 2018) and is most impacted by PK variability. To reduce the impact the sensitivity RIF has to PK variability, we simulated HRZE treatment while doubling the RIF dose. Indeed, we did observe faster granuloma sterilization and more complete sterilization in high-CFU, low PK granulomas, yet some granulomas in that group still failed to sterilize. Additionally, there was only a slight improvement in sterilization times for granulomas that were already easy to treat, indicating there might only be a modest improvement in treatment for a subset of those granulomas. Understanding an individual's PK profile for different drugs would be an important step in developing a personalized medicine approach to treatment.

While our model can recapitulate key experimental observations and also predict TB treatment outcomes, there are several limitations to our findings. Clinical results measure outcomes at the host level, and *GranSim* fundamentally simulates treatment and sterilization at the granuloma scale. The relevance of our results relies on the assumption that treatment at the granuloma scale is indicative of treatment at a host scale. Our model simulates primary granulomas and does not fully capture the full complexity of multiple pulmonary lesions as is observed during TB disease. It is appreciated that non-replicating and persisting Mtb are critical targets to achieve full sterilization of lesions, and while we observe this in our model, their importance could be amplified in cavitary disease or fibrotic lesions that are not captured in our model. Further, directly relating *in vitro* antimicrobial activity to *in vivo* efficacy does not necessarily capture the full range of antimicrobial activity that occurs within granulomas and may partially account for any discrepancies between our simulation results and clinical observations. An additional limitation of our model is that it currently assumes there are no interactions occurring between antibiotics, and synergistic or antagonistic combinations may be relevant in determining regimen efficacy (Swaminathan et al., 2016; Ma et al., 2019). Going forward, we are currently introducing synergistic and antagonistic antibiotic interactions to improve the PD model and further refine our estimates and predictions of granuloma sterilization (Chandrasekaran et al., 2016; Cokol et al., 2017; Cokol et al., 2018). The current model also does not include the development of antibacterial resistance, which may profoundly impact granuloma sterilization; see (Pienaar et al., 2018) for a previously published model examining development of resistance and a discussion of modeling resistance development. Finally, this work drew on data sets from a variety of human and animal studies, and predictions of treatment efficacy for other and newer drugs is dependent on the acquisition of similar data sets.

The significant impact that population PK variability and granuloma heterogeneity have on granuloma sterilization highlights the continued need for new approaches and drugs for treatment, and optimization of new regimens. Close collaboration between wet lab and computational scientists will help facilitate the evaluation of these new approaches and provide a more efficient and comprehensive development of new ways to treat TB.

## Data Availability Statement

The datasets generated for this study are available on request to the corresponding author.

## Author Contributions

The modeling and simulations of this work were completed by JC. VD provided assistance in pharmacokinetic and pharmacodynamic modeling, as well as MALDI-MSI. All authors (JC, VD, DK, and JL) contributed to the conceptualization of this work, analysis of results, and writing and editing.

## Funding

This research was supported by the following grants from the National Institutes of Health: U01HL131072 (to DK, JL, and VD), R01AI123093 (to DK), R01AI106398, and 1S10OD018072-01A1 (to VD). Simulations used resources of the National Energy Research Scientific Computing Center, which is supported by the Office of Science of the U.S. Department of Energy under Contract No. ACI-1053575 and the Extreme Science and Engineering Discovery Environment (XSEDE), which is supported by the National Science Foundation grant MCB140228.

## Conflict of Interest

The authors declare that the research was conducted in the absence of any commercial or financial relationships that could be construed as a potential conflict of interest.
