# Supplementary material for: Both Pharmacokinetic Variability and Granuloma Heterogeneity Impact the Ability of the First-Line Antibiotics to Sterilize Tuberculosis Granulomas
Source: Front Pharmacol. 2020 Mar 24;11:333. doi: 10.3389/fphar.2020.00333 (PMC7105635; doi:10.3389/fphar.2020.00333)
Supplement: Supplementary file 1 [file DataSheet_1.docx]

Supplementary Material


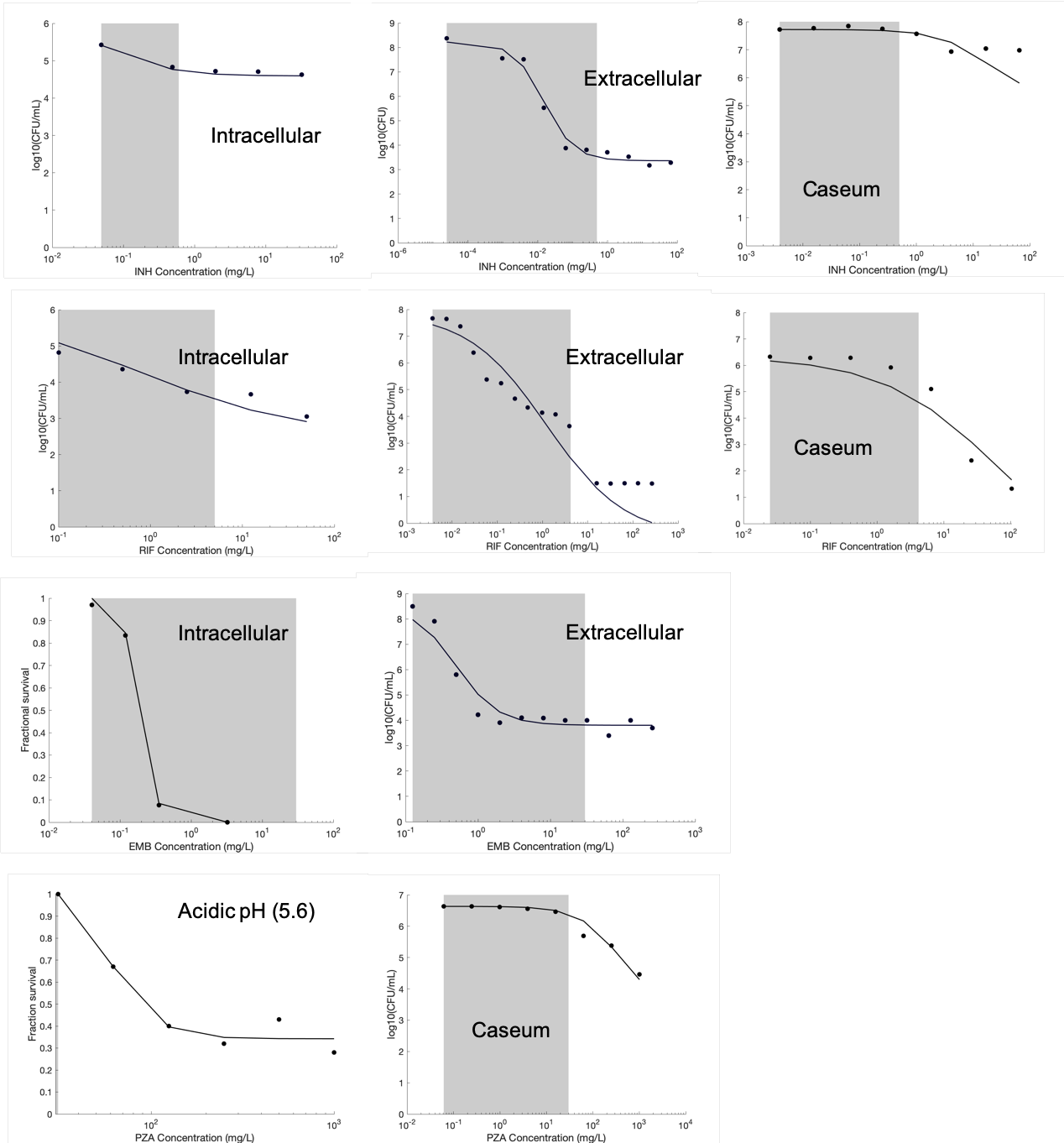


Supplementary Figure 1. Calibrated dose-response curves (lines) to *in vitro* data (dots) of dose response assays from individual experiments from the literature for each antibiotic under various culture conditions. References for the data sets are listed in Table 3. Calibrated curves for antibiotics are presented in rows in the order INH, RIF, EMB and PZA from top to bottom. Gray shaded regions indicate concentration values reached in granulomas.

Supplementary Figure 2. The simulated spatial distribution of RIF in a granuloma with larger amounts of caseum. Concentrations shown are based on those observed after six hours following a fifth daily dose of 10 mg/kg RIF. The granuloma is simulated on a 200x200 simulation grid representing 4 mm x 4 mm of lung tissue. Compared to the single-dose RIF concentration in a smaller granuloma, accumulation of RIF is observed in caseated regions of the granuloma (lighter pockets dispersed throughout the center of the granuloma).


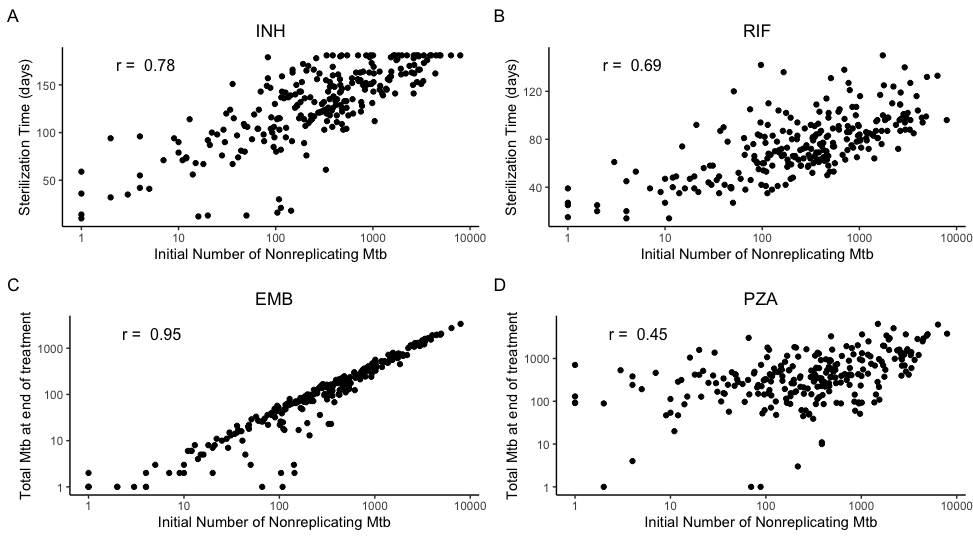


Supplementary Figure 3. The number of non-replicating Mtb at the beginning of therapy (300 days post infection) is positively correlated with sterilization time or number of remaining Mtb at the end of treatment (180 days of treatment). (INH, A; RIF, B; EMB, C; PZA, D). INH and EMB show strong positive relationships with correlation coefficients 0.78 and 0.95 respectively. The correlations are for RIF and PZA, with coefficients of 0.69 and 0.45 respectively. RIF has the best ability to kill non-replicating Mtb, so the sterilization time is less dependent on that subpopulation’s size. PZA shows little sterilizing activity in granulomas, so the number left at the end of treatment is more correlated with the initial number of Mtb.


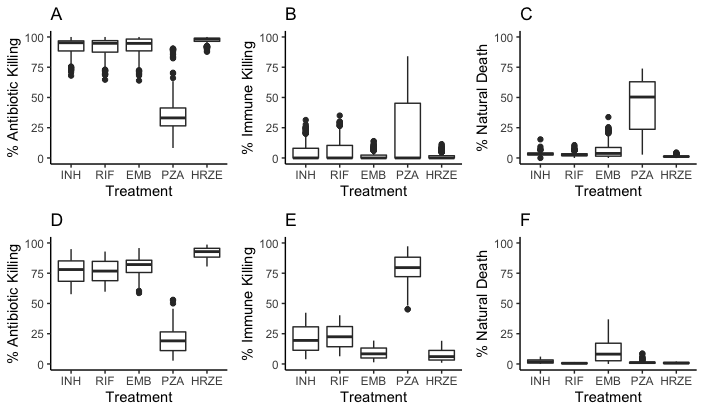


**Supplementary Figure 4.** The type of bacterial death during each treatment with average PK for low-CFU (A, B and C) and high-CFU (D, E, and F) granulomas. Figure shows the distribution of the percentrage of bacteria that die due to various causes over all granulomas. The types of death displayed are bacteria that died due to antibiotics (A and D), bacteria that died due to an immune response (B and E), and bacteria that died due to natural death through either starvation or death in caseum (C and F).


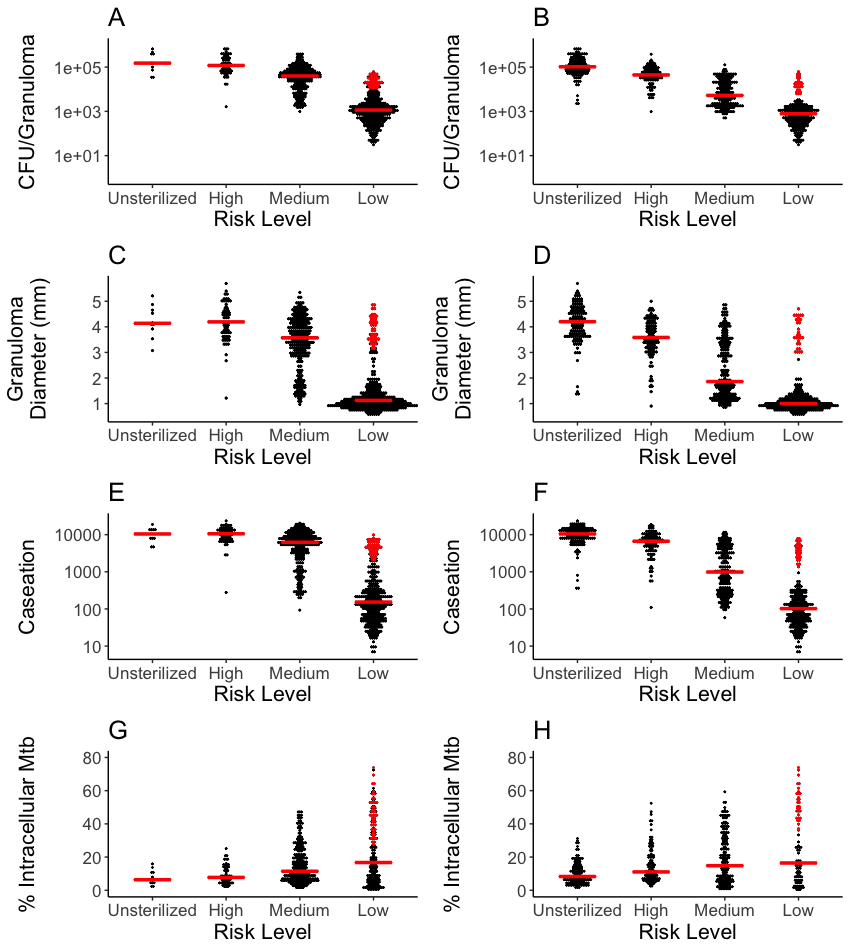


**Supplementary Figure 5.** Risk of granuloma treatment failure. Simulated granulomas were classified into four risk of treatment failure categories based on sterilization time when treated with HRZE with average (A, C, E, G) and low (B, D, F, H) PK exposure. The four categories were unsterile (failed to sterilize), high (sterilized after 150 days), medium (sterilized between 90 and 150 days), and low (sterilized before 90 days). The plots show the distribution of pretreatment CFU/granuloma, diameter, amount of caseation (measured in number of caseated compartments in the simulation), and percentage of intracellular Mtb, for each risk category, with the median value indicated by the red line. Each dot is a single granuloma. Red dots indicate granulomas that are low risk but have characteristics similar to unsterilized or high-risk granulomas.
